# *Fusobacterium nucleatum* Promotes the Progression of Colorectal Cancer Through Cdk5-Activated Wnt/β-Catenin Signaling

**DOI:** 10.3389/fmicb.2020.545251

**Published:** 2021-01-06

**Authors:** Xiang Li, Jiepeng Huang, Tingting Yu, Xiaoting Fang, Liqin Lou, Shijun Xin, Ling Ji, Feizhao Jiang, Yongliang Lou

**Affiliations:** ^1^Wenzhou Key Laboratory of Sanitary Microbiology, Key Laboratory of Laboratory Medicine, Ministry of Education, School of Laboratory Medicine and Life Sciences, Wenzhou Medical University, Wenzhou, China; ^2^Colorectal Cancer Research Center, Wenzhou Medical University, Wenzhou, China; ^3^The First Affiliated Hospital of Wenzhou Medical University, Wenzhou, China

**Keywords:** *Fusobacterium nucleatum*, Cdk5, Wnt/β-catenin signaling, colorectal cancer, migration

## Abstract

**Background/Aims:**

Growing evidence supports the direct link of *Fusobacterium nucleatum* with colorectal cancer (CRC). However, to date, the underlying mechanism of action remains poorly understood. In this study, we examined the effects of *F. nucleatum* on the progression of CRC and investigated whether cyclin-dependent kinase 5 (Cdk5) is involved in the effect through activating the Wnt/β-catenin signaling pathway.

**Materials and Methods:**

CRC tissues and matched histologically normal specimens were collected from patients who were diagnosed with CRC and underwent surgical treatment in our hospital between January 2018 and January 2019. Two human CRC cell lines, including DLD-1 and SW480, were utilized mainly for *in vitro* mechanistic investigations.

**Results:**

The abundance of *F. nucleatum* was significantly greater in CRC tissues than in cancer-free specimens, which was significantly correlated with the progression of CRC. *In vitro* investigations revealed that *F. nucleatum* significantly enhanced the proliferation and migration of CRC cells. Furthermore, *F. nucleatum* significantly induced the expression of Cdk5 and activation of the Wnt/β-catenin signaling pathway. Notably, knockdown of Cdk5 significantly abrogated the effects of *F. nucleatum* on cellular processes and Wnt/β-catenin signaling in relation to the progression of CRC.

**Conclusion:**

The results of this study demonstrate that *F. nucleatum* orchestrates a molecular network involving the direct role of Cdk5 in activating Wnt/β-catenin signaling to modulate CRC progression. Thus, in-depth investigations of *F. nucleatum-*associated molecular pathways may offer valuable insight into the pathogenesis of CRC, which may help further the development of treatment for this disease.

## Introduction

Colorectal cancer (CRC) is among the most common malignancies in both males and females worldwide (Torre et al., 2015). Although substantial progress has been made in the early diagnosis and treatment, the mortality rate of CRC remains high mainly due to frequently occurring drug resistance as well as the different phenotypes associated with genetic variants (Donna et al., 2012; Kocarnik et al., 2015). Therefore, there is an urgent need to better understand the pathogenesis of CRC and find a more effective treatment for this disease.

Recent studies have demonstrated that intestinal microbes play an important role in the development of CRC (Rowland, 2009; Wang et al., 2012; Zhu et al., 2013). In adults, the number of intestinal microbes can be as high as 10^14^, which is approximately 10 times higher than the number of human cells in total. There are 1,000 species of bacteria in the human intestine, and the number of encoded genes is 150 times greater than that of human genes (Qin et al., 2010; Zou et al., 2018). Recently, advancements in DNA/RNA sequencing technology have allowed researchers to gain a better understanding of the composition and distribution of intestinal microbes. Zhang et al. (2019) used quantitative PCR (qPCR) and bacterial 16S ribosomal RNA (rRNA) sequencing to show that the abundance of *Fusobacterium nucleatum* was significantly higher in both the tumor tissues and fecal specimens of CRC patients compared with healthy controls. Mima et al. (2016) evaluated the predictive value of *F. nucleatum* for the prognosis of CRC in 1,069 CRC patients, suggesting that the higher abundance of *F. nucleatum* was significantly associated with shorter survival in patients with CRC. Notably, *F. nucleatum* promoted tumor growth by activating the Wnt signaling pathway in CRC, suggesting the direct link of *F. nucleatum* with the development and progression of CRC (Mima et al., 2016). To date, the mechanism underlying the association remains unknown.

Cyclin-dependent kinases (Cdks) represent a class of serine/threonine protein kinases involved in regulating various cellular processes such as the cell cycle, transcription initiation, and control of certain metabolic cascades (Wilkaniec et al., 2016). In addition, it is worth noting that Cdk5, a member of the Cdk family, was originally identified in neuronal cells and plays a pivotal role in neuronal development, differentiation, and neurite outgrowth (Lew et al., 1992; Dhavan and Tsai, 2001). Recently, it has been shown that Cdk5 expression is altered during the cell cycle (Zhang et al., 2012; Nagano et al., 2013), suggesting a new regulatory role for Cdk5 in cell proliferation. Furthermore, it has been evident that Cdk5 is associated with a broad spectrum of diseases such as diabetes (Choi et al., 2010) and inflammation (Pareek et al., 2010; Berberich et al., 2011). There has been growing and important evidence showing that Cdk5 is also linked to a variety of cancers including prostate cancer, medullary thyroid carcinoma, liver cancer, lung cancer, and CRC (Cao et al., 2015; Zhang et al., 2015; Dorand et al., 2016; Herzog et al., 2016; Wei et al., 2016). However, the precise role of Cdk5 in the progression of CRC, such as invasion and metastasis, is still unclear.

In this study, we determined the effects of *F. nucleatum* on the progression of CRC and investigated the mechanisms whereby *F. nucleatum* exerts its effects on CRC. The findings of this study may provide a better understanding of the pathogenesis of CRC, thereby facilitating the development of more effective therapeutic approaches.

## Materials and Methods

### Study Subjects

A total of 84 CRC patients were prospectively enrolled between June 2018 and January 2019 in the First Affiliated Hospital of Wenzhou Medical University (Zhejiang, China). The patients were diagnosed with CRC and admitted to our hospital for surgical treatment. Surgically resected CRC tissues, histologically normal tissues adjacent to the tumor, and fecal specimens were obtained from the study patients. CRC was diagnosed and confirmed according to the clinical and histopathological findings. Tumor staging of CRC patients was conducted based on the Union Internationale Contre le Cancer tumor-node-metastasis staging system. Pathological classification of CRC was assessed according to the World Health Organization criteria. All fresh tissues were surgically resected, taken to the laboratory within 30 min, and cut into two pieces: one was formalin-fixed and paraffin-embedded for subsequent fluorescence *in situ* hybridization (FISH) analysis and immunohistochemistry (IHC), whereas the other one was used for Western blot (WB) analysis of protein levels. In July 2018, 90 fecal samples were taken from healthy donors as normal controls. Total fecal DNA was extracted using a fecal DNA extraction kit (Hangzhou Guhe Biotechnology Co., Ltd., Hangzhou, China) according to the manufacturer’s protocol. The V4 region of the bacterial 16S rRNA marker gene (16Sv4) was PCR-amplified and sequenced by Hangzhou Guhe Biotechnology Co., Ltd. This study was reviewed and approved by the Ethics Committee of the First Affiliated Hospital of Wenzhou Medical University. Written informed consent was obtained from each patient prior to the study.

### Bacteria Strains

*F. nucleatum* strain ATCC 25586 was purchased from American Type Culture Collection (ATCC, Manassas, VA). *Escherichia coli* strain DH5α was obtained from Tiangen Biotech (Beijing, China). *F. nucleatum and* DH5α were cultured according to the manufacturers’ instructions. For treatment, CRC cells were cocultured with *F. nucleatum* at multiplicities of infection (MOIs) of 50 and 100 or DH5α at MOIs of 50 and 100 for 24 h.

### Colorectal Cancer Cell Lines and Cell Culture

CRC cell lines, including DLD-1 and SW480, were obtained from ATCC. DLD-1 and SW480 cells were maintained and grown in Dulbecco’s Modified Eagle Medium (DMEM) supplemented with 10% fetal bovine serum (FBS) (Invitrogen, Waltham, MA, United States), 100 μg/ml streptomycin (Sangon, Shanghai, China), and 100 U/ml penicillin G (Sangon) in a 37°C incubator containing 5% CO_2_.

### Transfection, Infection, and Stable Expression of Cyclin-Dependent Kinase 5-Specific shRNA

Cdk5-specific short hairpin RNAs (shRNAs) (#1, TRCN0000021465; #2, TRCN0000199652) were obtained from Sigma-Aldrich (Darmstadt, Germany). VSV-G-pseudotyped lentiviral vectors were produced in 293T cells by co-transfecting 293T cells (6 × 10^6^) with Cdk5-specific shRNAs or control using Lipofectamine 2,000 (Invitrogen). The packaging system used was as follows: pMDLg/RRE (1 pmol), pMD2.G (0.5 pmol), and pRSV-Rev (0.5 pmol) in accordance with the manufacturer’s instructions. At 48 and 72 h post-transfection, the supernatant was removed and filtered through a cellulose acetate membrane (0.45 μm pore size). DLD-1 and SW480 cells were seeded in a six-well plate at 1 × 10^5^ cells/well and infected with lentivirus (100 μl per plate) for 24 h. Cells stably expressing lentiviral Cdk5-shRNA were selected using puromycin (1 mg/ml) for 3–4 weeks.

### Cell Viability

The 3-(4,5-dimethylthiazol-2-yl)-2,5-diphenyltetrazolium bro- mide (MTT) cell viability assay was conducted as previously described (Mosmann, 1983). Briefly, cells were placed in a 24-well plate (1 × 10^4^ per well) and subsequently incubated with *F. nucleatum* (MOI of 100:1) for 5 days or treated with phosphate-buffered saline (PBS) as a negative control. Cells were incubated with 20 μl MTT (5 mg/ml) for 3 h, after which they were lysed with 100 μl dimethyl sulfoxide. Cell viability was assayed on a plate reader at a wavelength of 570 nm (BioTek Instruments, Winooski, VT, United States).

### Colony Formation Assay

CRC cells were used for the colony formation assay. After incubation at 37°C, 5% CO_2_ incubator for 2 weeks, cells were fixed in paraformaldehyde and stained with crystal violet. The colony formation rate was examined, and the following formula was used for its calculation: rate of colony formation (%) = the number of colonies/seeded cells × 100%.

### Invasion Assays

The invasion assay was performed as previously described (Wu et al., 2011). In brief, 100 μl Matrigel (1:30 dilution) was initially added to the Transwell polycarbonate filter. CRC cells were trypsinized, washed with DMEM supplemented with 1% FBS, and resuspended in DMEM/1% FBS (5 × 10^5^ cells/ml). Then, 100 μl cell suspension was seeded, and subsequently, 600 μl DMEM supplemented with 10% FBS was added to the lower chambers. After the cells were incubated in a CO_2_ incubator for 12 h, they were fixed, stained, and quantified as previously described (Wu et al., 2011).

### Cell Migration Assay

A scratch wound healing assay was conducted for examination of cell migration. After DLD-1 or SW480 cells (5 × 10^5^ per well) reached 100% confluence, a wound gap was created by scratching the resultant monolayer using a 10 μl pipette tip, which was visualized under an inverted phase-contrast microscope. The rate of wound closure was calculated using the following formula: Wound closure (%) = [(Original wound area–Open area on at time point)/Original wound area] × 100%.

### Cell Cycle Analysis

Flow cytometry was performed for cell cycle analysis using propidium iodide (PI). Briefly, CRC cells were placed in a six-well plate (1 × 10^5^ cells per well) and incubated at 37°C, 5% CO_2_ incubator. After 12 h, the cells were harvested, and then the CycleTest Plus DNA Reagent kit (BD Biosciences, San Jose, CA, United States) was used for PI staining in accordance with the manufacturer’s manual. The number of cells at various stages of cell cycle, including G0/G1, S, and G2/M stages, was counted, and the proportion was calculated.

### Immunohistochemical Staining

IHC was conducted on sections (4 μm in thickness) of paraffin-embedded, formalin-fixed human CRC tissues and matched with histologically normal tissues adjacent to the tumor, following a protocol as previously described (Liu et al., 2014). In brief, tissue microarray (TMA) slides were baked at 60°C for 1 h, deparaffinized, dehydrated, treated in citrate buffer (pH 6.0), blocked using equine serum, and incubated with 3% hydrogen peroxide. Subsequently, TMA slides were incubated with anti-Cdk5 antibody (1:25 dilution) at 4°C overnight, followed by incubation with horseradish peroxidase (HRP)-conjugated goat anti-rabbit secondary antibody (dilution, 1:2,000) at 37°C for 1 h. Then the slides were stained with hematoxylin, dehydrated, cleared, and mounted. Cdk5 was identified with ImagePro Plus software 6.0 from Media Cybernetics (Rockville, MD, United States). The proportion (P) of positive cells was assessed as the percentage of cell stained: 0 (0%), 1 (1–25%), 2 (26–50%), 3 (51–75%), or 4 (76–100%) under an Eclipse 50i/55i optical microscope. Staining intensity (I) was quantified by assigning scores as follows: 0 (none), 1 (weak), 2 (moderate), or 3 (strong). A previously described formulation was used for examination of histological grade (H-score): H-score = Σ (I × P) (Xu et al., 2016). The score of Cdk5 expression was calculated based on the value of the percent positivity score multiplied by the staining intensity score as: score as “–” (score, 0–1), “ + ” (score, 2–3), “ + + ” (score, 4–5), and “ + + + ” (score ≥ 6).

### Immunofluorescence Assay

DLD-1 or Cdk5-knockdown DLD-1 cells (5 × 10^4^ cells per well) were seeded in a 24-well plate. Immunofluorescence assay was performed in cells with different treatments. Briefly, the cells were fixed in 2% paraformaldehyde, permeabilized in 0.5% Triton X-100, and treated with 10% goat serum. The permeabilized cells were subsequently treated with β-catenin monoclonal antibody, washed with PBS, and then incubated with Cy3-labeled goat anti-rabbit IgG secondary antibody (1:100). Cells were incubated with 4′,6-diamidino-2-phenylindole (DAPI) staining solution (Beyotime Biotechnology, Shanghai, China). After washing with PBS, the cells were visualized under the Olympus Fluoview FV1000 confocal laser scanning microscope (Olympus, Tokyo, Japan).

### Fluorescence *in situ* Hybridization Analysis

FISH analysis was conducted to examine the abundance of *F. nucleatum* using a specific probe. In brief, sections of formalin-fixed, paraffin-embedded tissue were cut into sections 5 μm thick and hybridized in accordance with the manufacturer’s instructions (FOCOFISH, Guangzhou, China). The following universal bacterial probe (EUB338; Cy3-labeled) was used: 5′-GCT GCC TCC CGT AGG AGT-3′. The following probe specific to *F. nucleatum* (FUS664; FITC-labeled) was used: 5′-CTT GTA GTT CCG C(C/T) TAC CTC-3′. The resulting slides were visualized and examined under a fluorescence microscope (BX53F; Olympus); five random fields per sample (200 × magnification) were examined, and the average number of bacteria per field was calculated. The abundance of *F. nucleatum* was classified into three categories: negative (< 5), low (5–20), high (> 20). For other bacteria, positive status was defined as > 5 bacteria per field with the EUB 338 probe, whereas negative status was determined with the FUS664 probe.

### Quantitative PCR Reaction

qPCR was conducted to examine RNA expression. Briefly, total RNA was extracted, and cDNA was synthesized on the Applied Biosystems 7,500 Real-Time PCR System (Applied Biosystems, Foster City, CA, United States), and target gene expression was analyzed using the 2^–ΔΔCt^ method as previously described (Livak and Schmittgen, 2001). Glyceraldehyde-3-phosphate dehydrogenase (GAPDH) and 16S rDNA served as internal reference transcripts. The primers are listed in Supplementary Table 1. The thermocycling conditions were summarized as follows: 20 s at 95°C, 40 cycles of 5 s at 95°C, followed by 30 s at 60°C.

### Western Blot Analysis

WB analysis was conducted to determine the protein expression in human tissues and colorectal cells. In brief, total protein was extracted, and concentration was measured using the bicinchoninic acid (BCA) protein assay. Total protein (20 μg) was initially separated on 12% sodium dodecyl sulfate polyacrylamide gel electrophoresis and transferred to a nitrocellulose membrane. After blocking in 5% milk, membranes were incubated with specific primary antibodies (1:1,000 dilution) including Cdk5, β-catenin, cyclin D1, c-Myc, cyclin E1, cyclin A, total signal transducer and activator of transcription 3 (STAT3), phosphorylated STAT3 (p-STAT3), β-actin, GAPDH, and tubulin at 4°C overnight. Membranes were subsequently incubated with HRP-conjugated secondary antibody (Beyotime Institute of Biotechnology). Tubulin and GAPDH served as the loading controls. Levels of protein were quantified using ImageJ 1.43 u/Java 1.6.0–10 from the National Institutes of Health (Bethesda, MD, United States).

### Xenografts and Animal Experiments

Twenty BALB/c nude mice (4–6 weeks) were used in the animal studies. The study involving experimental animals was approved by the Institutional Animal Committee of Wenzhou Medical University. BALB/c nude mice were injected subcutaneously with 1 × 10^6^ DLD-1 cells per mouse. Tumor size in diameter and body weight were measured three times per week, with tumor size measured with calipers daily or every other day. The following formula was used for calculation of tumor volumes: volume = (width)^2^ × length/2.

### Statistical Analysis

Statistical analysis was conducted using SPSS 19.0 (SPSS, Chicago, IL, United States). The potential association between the abundance of *F. nucleatum* and mRNA expression was assessed by linear regression. Data are expressed as the mean ± standard deviation. One-way analysis of variance (ANOVA) was used for between-group comparisons, whereas comparison among multiple groups was made using ANOVA and the least significant difference test. *P* < 0.05 was considered statistically significant.

## Results

### Upregulation of Cyclin-Dependent Kinase 5 Expression and Its Correlation With the Abundance of *F. nucleatum* in Colorectal Cancer

**FIGURE 1:**
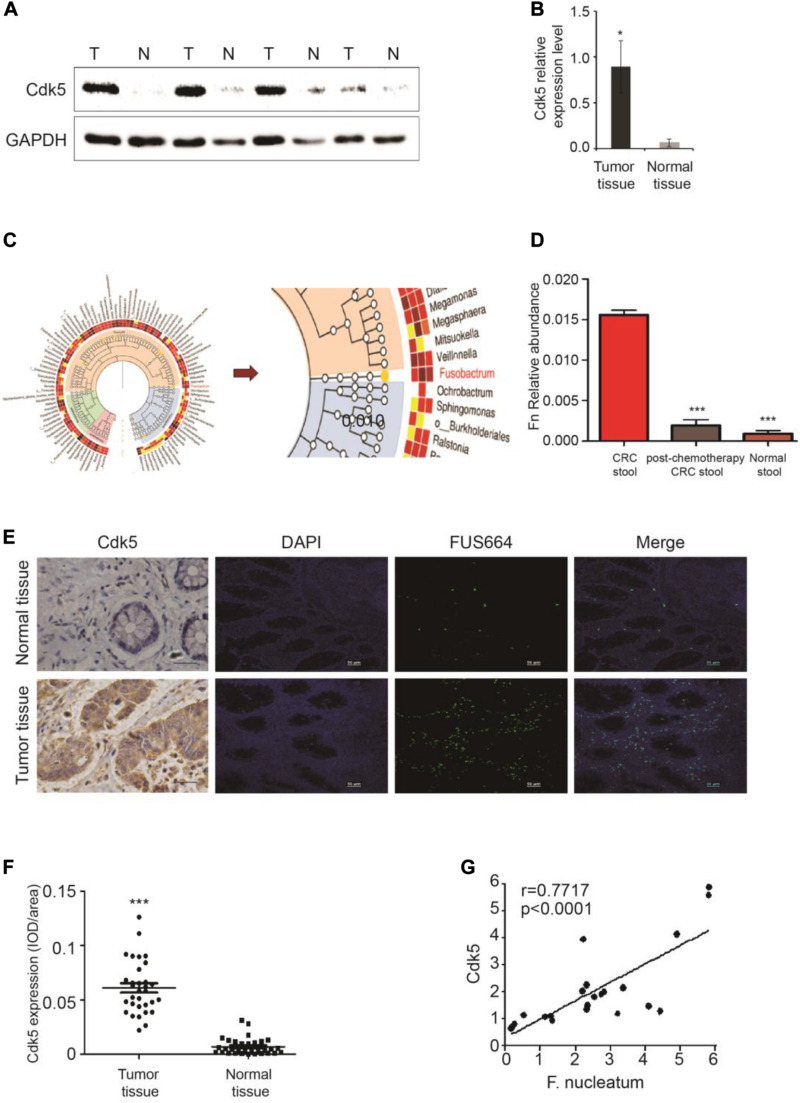
Upregulation of cyclin-dependent kinase 5 (Cdk5) protein expression and its correlation with Fusobacterium nucleatum in colorectal cancer (CRC). **(A)** Cdk5 protein levels were detected by Western blot (WB) analysis in CRC and normal tissues. **(B)** Statistical analysis of Cdk5 mRNA expression in **(A)** (*P < 0.05). **(C,D)** *Fusobacterium* abundance was significantly higher in CRC tissues in comparison with controls. **(E–G)** The abundance of *F. nucleatum* was positively correlated with Cdk5 expression in tumor tissues. **(E)** Representative images showing that the abundance *of F. nucleatum* (green dot) in tumor tissues is associated with high expression of Cdk5. **(F)** Immunohistochemical scatter statistics of Cdk5 protein levels in 21 CRC tissues and normal tissues from patients (***P < 0.001, 200 × magnification). **(G)** Statistical analysis of Cdk5 and *F. nucleatum* mRNA expression in 18 tumor tissues from patients and 18 healthy controls (*r* = 0.7717, *P* < 0.0001; two-tailed, non-parametric Spearman’s correlation).

We initially examined Cdk5 protein expression in the surgically resected CRC tissues and matched adjacent histologically cancer-free specimens from each patient. WB analysis indicated that Cdk5 protein expression in CRC tissues was 14 times greater than that in the normal specimens (*P* < 0.05; Figures 1A,B). DNA sequencing revealed significant differences in the abundance of microbiota between CRC and post-chemotherapy CRC stool and histologically normal control stool samples, with a predominance of *Firmicutes*, *Proteobacteria*, and *Fusobacterium*. Among all *Firmicutes* species, the abundance of *c-clostridiales* as well as *Erysipelotrichaceae* was significantly lower in CRC and post-chemotherapy CRC stool compared to normal control stool samples. In the *Proteobacteria* species, the abundance of *Pseudomonas* was significantly higher in CRC stool than in normal control stool samples, whereas the relative abundance of *Fusobacterium* was lower in CRC stool than in post-chemotherapy CRC stool samples (Figures 1C,D). Furthermore, we found that the abundance of *F. nucleatum* was positively correlated with Cdk5 mRNA levels (*r* = 0.7717, *P* < 0.0001; Figure 1G). Next, FISH analysis was performed to examine *F. nucleatum* richness as well as Cdk5 expression in the tissue samples obtained from CRC patients. As shown in Figures 1E,F, a greater abundance of *F. nucleatum* in CRC tissues was significantly associated with higher levels of Cdk5 (*P* < 0.001).

### *F. nucleatum* Directly Upregulates Cyclin-Dependent Kinase 5 Expression and Promotes Cell Proliferation and Migration

**FIGURE 2:**
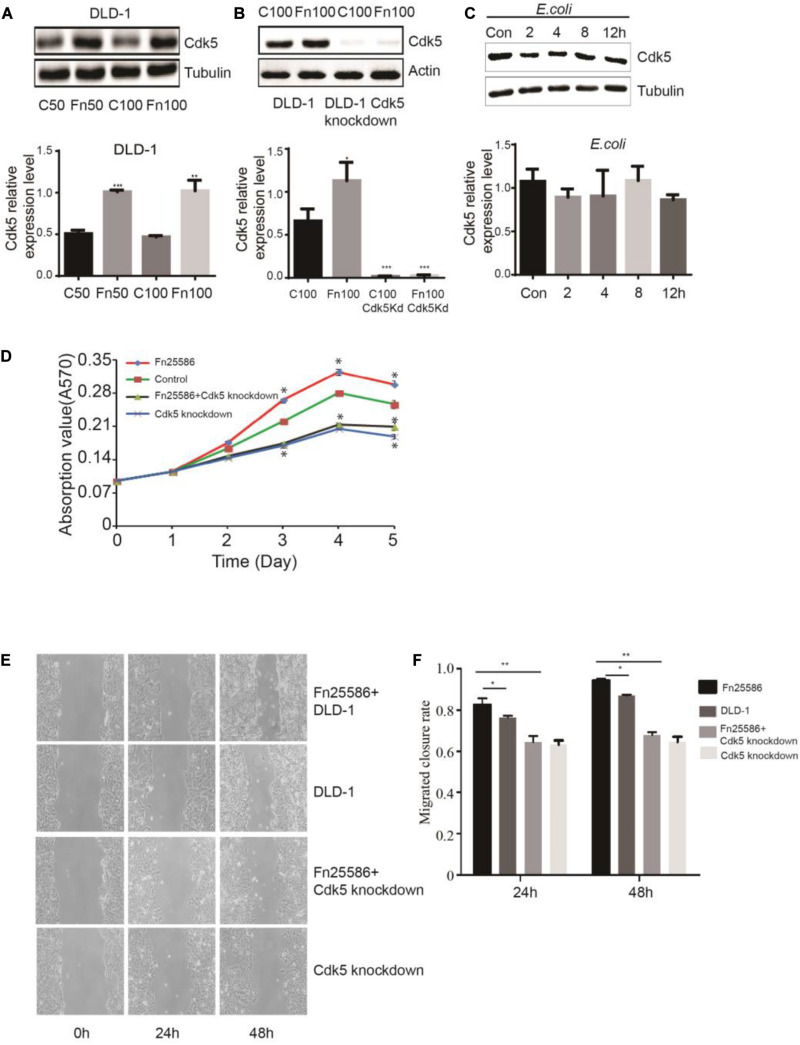
*Fusobacterium nucleatum* regulates DLD-1 cancer cell proliferation through upregulated cyclin-dependent kinase 5 (Cdk5) expression. **(A)** The protein levels and quantification of Cdk5 in DLD-1cells treated with *F. nucleatum* were measured by Western blot (WB) analysis. Data are expressed as mean ± SEM from at least three independent experiments. ***P < 0.001 and **P < 0.01 vs. control. **(B)** The protein levels and quantification of Cdk5 in DLD-1 cells and Cdk5-knockdown DLD-1 cells treated with *F. nucleatum* were measured by WB analysis. Data are expressed as mean ± SEM from at least three independent experiments. ***P < 0.001 and *P < 0.05 vs. control. **(C)** The protein levels and quantification of Cdk5 in DLD-1 cells cocultured with *Escherichia coli* (DH5α) over time were measured by WB analysis. Data are expressed as mean ± SEM from at least three independent experiments. **(D)** *F. nucleatum* can promote the proliferation of colorectal cancer cells through Cdk5. **(E)** Wound-healing assay in DLD-1 cells and Cdk5-knockdown DLD-1 cells, respectively, after treated with *F. nucleatum* (× 400). **(F)** Quantification of wound-healing assays. Data are expressed as mean ± SEM from at least three independent experiments. *P < 0.05 vs. DLD-1, **P < 0.01 vs. Cdk5-knockdown DLD-1.

**FIGURE 3:**
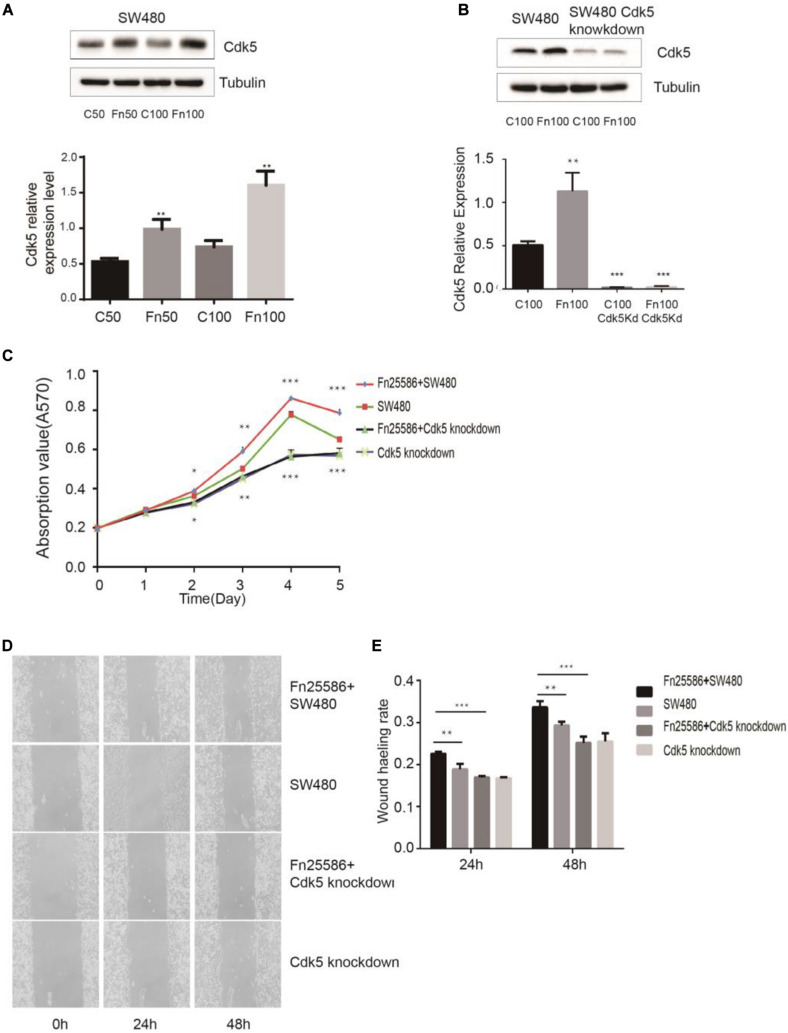
*Fusobacterium nucleatum* regulates SW480 cancer cell proliferation through upregulated cyclin-dependent kinase 5 (Cdk5) expression. **(A)** The protein levels and quantification of Cdk5 in SW480 cells treated with *F. nucleatum* were measured by Western blot (WB) analysis. Data are expressed as mean ± SEM from at least three independent experiments. **P < 0.01 vs. control. **(B)** The protein levels and quantification of Cdk5 in SW480 cells and Cdk5-knockdown SW480 cells treated with *F. nucleatum* were measured by WB analysis. Data are expressed as mean ± SEM from at least three independent experiments. ***P < 0.001 vs. control. **(C)** *F. nucleatum* can promote the proliferation of colorectal cancer cells through Cdk5. *P < 0.05, **P < 0.01, and ***P < 0.001 vs. SW480. **(D)** Wound-healing assay in SW480 cells and Cdk5-knockdown SW480 cells, respectively, after treated with *F. nucleatum* (× 400). **(E)** Quantification of wound-healing assays. Data are expressed as mean ± SEM from at least three independent experiments. **P < 0.01 vs. SW480, ***P < 0.001 vs. Cdk5-knockdown SW480.

To determine whether *F. nucleatum* can directly affect Cdk5 expression, CRC DLD-1 and SW480 cells were co-incubated with different concentrations (bacteria per cell) of *F. nucleatum* (strain ATCC 25586), corresponding to MOI of 100 or 50. After 48 h, Cdk5 protein expression was detected by WB analysis. As shown in Figures 2A,B, Cdk5 protein expression significantly increased in the DLD-1 cells in response to *F. nucleatum* inoculation. Knockdown of the Cdk5 gene in DLD-1 cells led to downregulation of Cdk5 protein levels, which remained significantly unchanged after *F. nucleatum* treatment; similar results were found in SW480 cells (Figures 3A,B). Notably, in DLD-1 cells cocultured with *E. coli* (DH5α), no significant alteration in Cdk5 protein expression was observed (Figure 2C). These data suggested that *F. nucleatum* directly enhanced Cdk5 expression. To determine whether *F. nucleatum* could regulate the proliferation of DLD-1 and SW480 cells and whether Cdk5 could play a role in the *F. nucleatum-*mediated effect, CRC cell lines (DLD-1 and SW480) and Cdk5-knockdown cells were inoculated with *F. nucleatum* 25,586 (MOI 100). As shown in Figure 2D, *F. nucleatum* inoculation resulted in a significant increase in the cell proliferation of wild-type DLD-1 cells but not in Cdk5-knockdown DLD-1 cells, suggesting the direct involvement of Cdk5 promoting *F. nucleatum-*mediated CRC cell proliferation. To further determine whether *F. nucleatum* exerted its effect on the proliferation of DLD-1 cells through Cdk5, a wound was generated in cells using a 10 μl pipette tip, and the wound healing, which was defined as the rate at which cells grew into the areas of the wound, was examined after 24 and 48 h (Figures 2E,F). After 24 h, the wound healing rate of the DLD-1 cells inoculated with *F. nucleatum* was the highest among all groups. Specifically, the rate of DLD-1 cells inoculated with *F. nucleatum* was significantly higher than DLD-1 cells without *F. nucleatum*, and Cdk5-knockdown DLD-1 cells with or without *F. nucleatum.* In Cdk5-knockdown DLD-1 cells, cell migration was significantly slower in the *F. nucleatum* group than in the control group (*P* < 0.01), suggesting that Cdk5 depletion reduced the migration ability of the colon cancer cells. At 48 h, the migration of DLD-1 cells co-incubated with *F. nucleatum* 25,586 was 1.09 times that of control DLD-1 cells and 1.43 times that of Cdk5-knockdown cell lines.

*F. nucleatum* exerting its effect on the proliferation of SW480 cells through Cdk5 is shown in Figures 3C–E. MTT results indicated that *F. nucleatum* inoculation significantly increased the proliferation of wild-type SW480 cells but not in Cdk5-knockdown SW480 cells. Wound healing results showed that the wound healing rate of SW480 cells inoculated with *F. nucleatum* was significantly higher than that of SW480 cells without *F. nucleatum* and Cdk5-knockdown SW480 cells with or without *F. nucleatum.* At 48 h, the migration of Cdk5-knockdown cells cocultured with *F. nucleatum* 25,586 was 0.85 times that of control SW480 cells and 0.74 times that of SW480 cells co-incubated with *F. nucleatum* 25,586. These results indicated that *F. nucleatum* promoted the migration of CRC cells through Cdk5.

### Cyclin-Dependent Kinase 5 Is Involved in the Activation of the Wnt/β-Catenin Signaling

**FIGURE 4:**
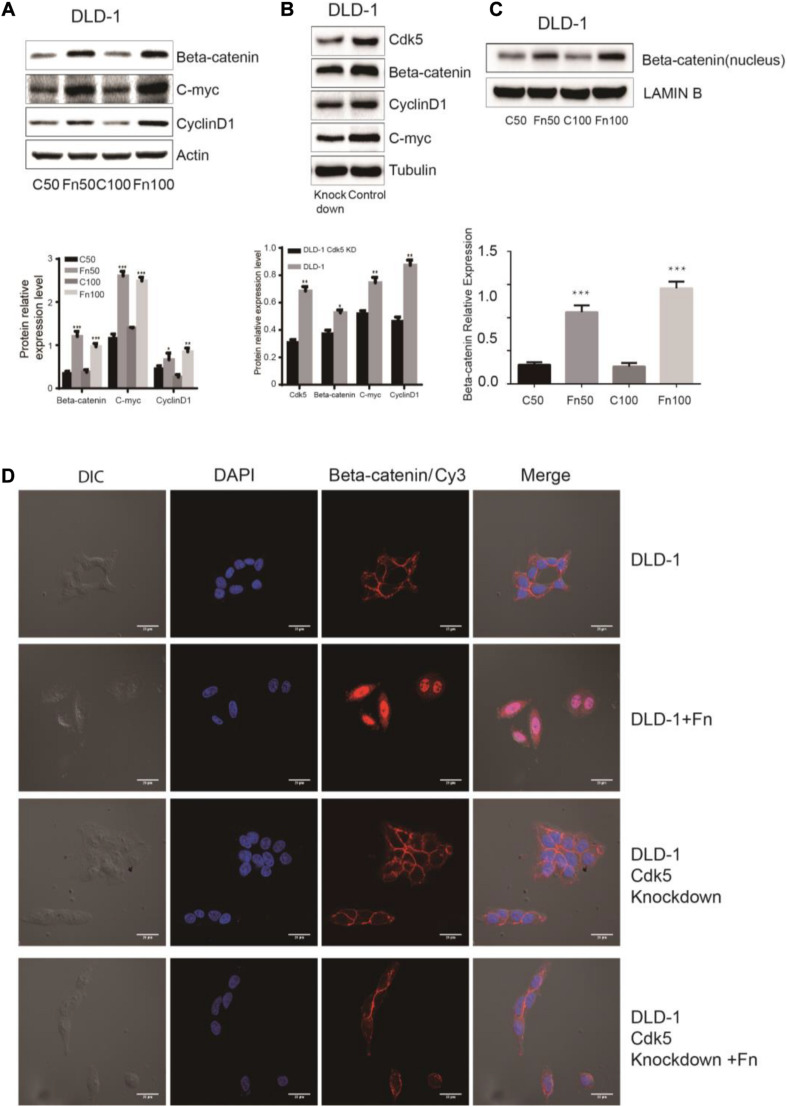
*Fusobacterium nucleatum* activates Wnt/β-catenin signaling and promotes the nuclear translocation of β-catenin through cyclin-dependent kinase 5 (Cdk5) in DLD-1 cell lines. **(A)** The expression levels and quantification of β catenin, c-Myc, and cyclin D1 treated with *F. nucleatum* were measured by Western blot (WB) analysis in DLD-1 cells. Data are expressed as mean ± SEM from at least three independent experiments. *P < 0.05, **P < 0.01, and ***P < 0.001 vs. control. **(B)** The expression levels and quantification of Cdk5, β catenin, c-Myc, and cyclin D1 were measured by WB analysis in DLD-1 and Cdk5-knockdown DLD-1 cells. Data are expressed as mean ± SEM from at least three independent experiments. *P < 0.05 and **P < 0.01 vs. control. **(C)** The expression levels and quantification of nuclear beta-catenin treated with *F. nucleatum* were measured by WB analysis in DLD-1 cells. Data are expressed as mean ± SEM from at least three independent experiments. ***P < 0.001 vs. control. **(D)** *F. nucleatum* affects β catenin cellular localization through Cdk5. Cells were seeded onto coverslips and treated with *F. nucleatum* or control for 12 h. The cells were then subjected to immunofluorescence staining. The cells were immunostained with an anti-β catenin (red fluorescence). The nucleus was stained with 4′,6-diamidino-2-phenylindole (DAPI) (blue fluorescence). Cy, cyanine; DIC, differential interference contrast.

**FIGURE 5:**
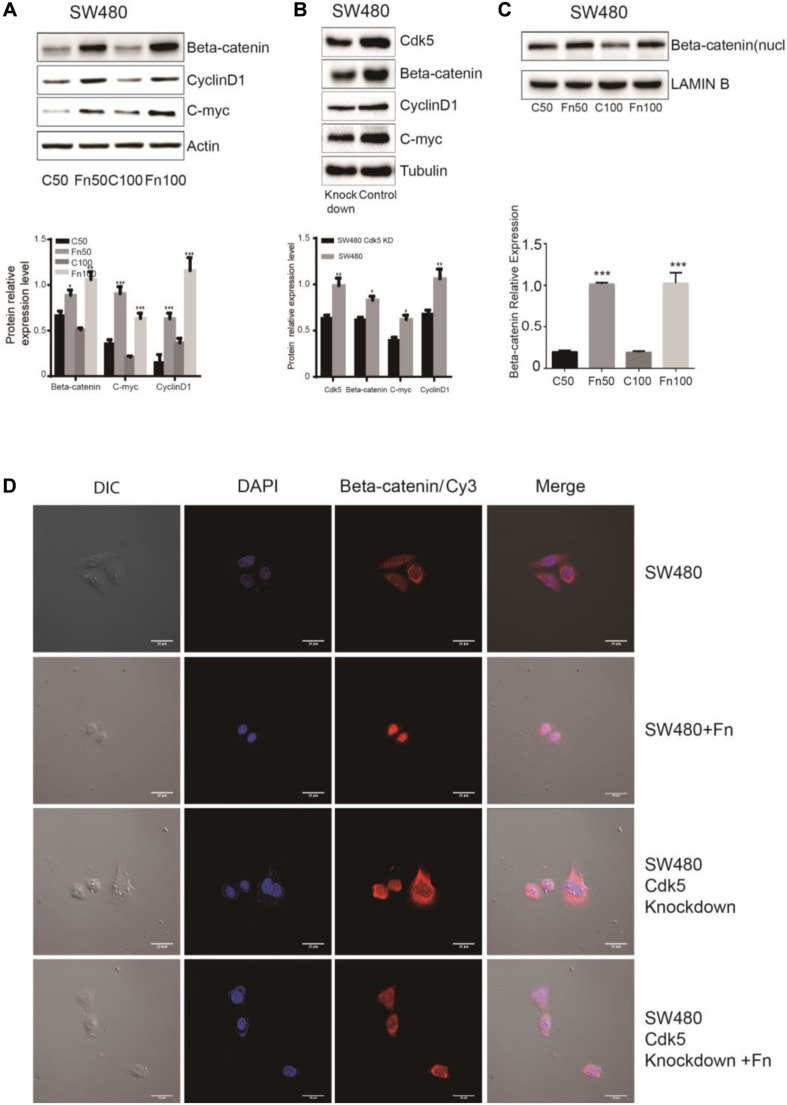
*Fusobacterium nucleatum* activates Wnt/β-catenin signaling and promotes the nuclear translocation of β-catenin through cyclin-dependent kinase 5 (Cdk5) in SW480 cell lines. **(A)** The expression levels and quantification of β catenin, c-Myc, and cyclin D1 treated with *F. nucleatum* were measured by Western blot (WB) analysis in SW480 cells. Data are expressed as mean ± SEM from at least three independent experiments. *P < 0.05, **P < 0.01, and ***P < 0.001 vs. control. **(B)** The expression levels and quantification of Cdk5, β catenin, c-Myc, and cyclin D1 were measured by WB analysis in SW480 and Cdk5-knockdown SW480 cells. Data are expressed as mean ± SEM from at least three independent experiments. *P < 0.05 and **P < 0.01 vs. control. **(C)** The expression levels and quantification of nuclear beta-catenin treated with *F. nucleatum* were measured by WB analysis in SW480 cells. Data are expressed as mean ± SEM from at least three independent experiments. ***P < 0.001 vs. control. **(D)** *F. nucleatum* affects β catenin cellular localization through Cdk5. Cells were seeded onto coverslips and treated with *F. nucleatum* or control for 12 h. The cells were then subjected to immunofluorescence staining. The cells were immunostained with an anti-β catenin (red fluorescence). The nucleus was stained with 4′,6-diamidino-2-phenylindole (DAPI) (blue fluorescence). Cy, cyanine; DIC, differential interference contrast.

The Wnt signaling pathway, characterized by accumulation in the cytoplasm and nuclear translocation of β-catenin, plays an important role in CRC. To test the hypothesis that *F. nucleatum* affects β-catenin localization, DLD-1 and SW480 cells were inoculated with *F. nucleatum* (MOI 50 or 100), and localization of β-catenin protein was analyzed by immunofluorescence staining and WB analysis. As shown in Figures 4D, 5D, β-catenin was localized in the nucleus in response to *F. nucleatum*, and there was a significant difference between cells treated with or without *F. nucleatum*. After Cdk5 knockdown, β-catenin cannot accumulate in the cell nucleus treated with or without *F. nucleatum*. WB analysis indicated that the expression levels of nuclear beta-catenin treated with *F. nucleatum* increased significantly (Figures 4C, 5C). In parallel, the expression levels of c-Myc and cyclin D1, two downstream genes in the pathway, were elevated in the *F. nucleatum*-treated cells compared to the controls (Figures 4A, 5A). It was of note, however, that the expression levels of β-catenin, c-Myc, and cyclin D1 were significantly decreased in Cdk5-knockdown cells (Figures 4B, 5B). These results indicated that *F. nucleatum* activated Wnt/β-catenin signaling *via* upregulation of Cdk5.

### *F. nucleatum* Promotes the Phosphorylation of STAT3 in Cells and Tissues

**FIGURE 6:**
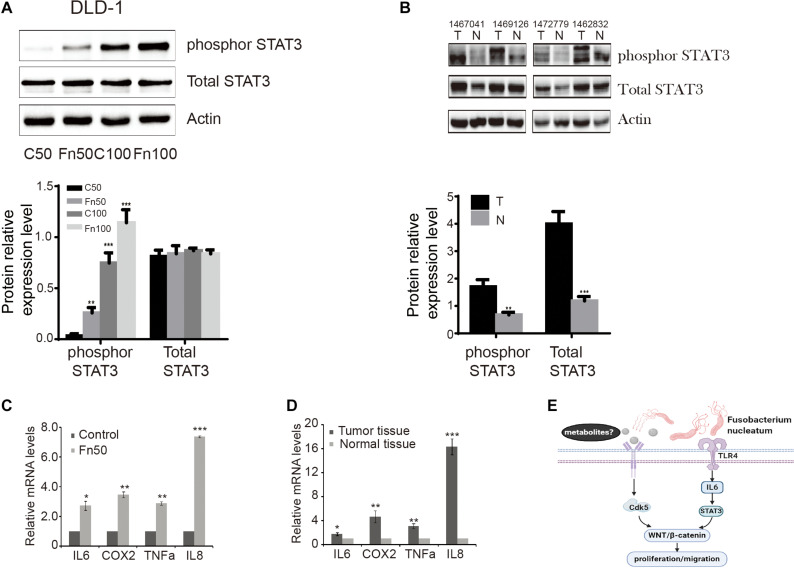
*Fusobacterium nucleatum* induces the phosphorylation of signal transducer and activator of transcription 3 (STAT3) in cells and tissues. **(A)** The expression levels and quantification of phosphorylated STAT3 and total STAT3 treated with *F. nucleatum* were measured by Western blot (WB) analysis in DLD-1 cells. Data are expressed as mean ± SEM from at least three independent experiments. ***P < 0.001 vs. control. **(B)** The expression levels and quantification of phosphorylated STAT3 and total STAT3 in tumor and normal tissues. Data are expressed as mean ± SEM from at least three independent experiments. **P < 0.01 and ***P < 0.001 vs. control. **(C)** Interleukin (IL)-6, cyclooxygenase 2 (COX-2), tumor necrosis factor alpha (TNF-α), and IL-8 mRNA levels in SW480 cell lines treated with Fn25586. Data are presented as mean ± SEM *P < 0.05, **P < 0.01, and ***P < 0.001 vs. control. *F* = 73.872. **(D)** IL-6, COX-2, TNF-α, and IL-8 mRNA levels in tumor and normal tissues. Data are presented as mean ± SEM *P < 0.05, **P < 0.01, and ***P < 0.001 tumor tissue vs. normal tissue. **(E)** Hypothetical model for *F. nucleatum*-mediated promotion of the proliferation and migration of colorectal cancer cells.

Phosphorylation of STAT3 leads to the modulation of various cellular processes, such as invasion and metastasis (Avalle et al., 2017). We determined the effects of *F. nucleatum* on p-STAT3 in DLD-1 cells. As shown in Figure 6A, levels of p-STAT3 protein were significantly higher in DLD-1 cells treated with *F. nucleatum* compared with control cells. Similarly, higher levels of p-STAT3 protein were found in CRC tissues compared with matched normal specimens (Figure 6B). Considering that interleukin 6 (IL-6) is a major activator of STAT3 (35), we also measured the mRNA levels of IL-6 in CRC cells with or without *F. nucleatum* incubation, as well as in CRC tissues and matched normal specimens. IL-6 mRNA levels were significantly greater in SW480 cells treated with *F. nucleatum* than in control cells, as well as in CRC tissues compared with adjacent normal specimens (Figures 6C,D). In parallel, we found that alterations in IL-8, cyclooxygenase 2 (COX-2), and tumor necrosis factor alpha (TNF-α) mRNA expression were similar to those of IL-6 in SW480 cells and human CRC tissues.

## Discussion

The major novel findings of this study can be summarized as follows. (1) The abundance of *F. nucleatum* was significantly higher in CRC stool samples than in matched histologically normal specimens. (2) *F. nucleatum* enhanced the proliferation and migration of CRC cells, and there was a significant correlation between *F. nucleatum* and progression of CRC. (3) *F. nucleatum* induced Cdk5 expression, and knockdown of Cdk5 significantly abrogated *F. nucleatum-*mediated effects. (4) Cdk5 activated the Wnt/signaling pathway, thereby promoting the proliferation and migration of CRC cells in response to *F. nucleatum*. Together, these findings suggested that *F. nucleatum* promoted the progression of CRC by activating the Cdk5-Wnt/signaling pathway.

Cdk5 is considered an atypical member of the Cdk family mainly due to its binding to non-cyclin proteins [e.g., p35 (CDK5R1) and p39 (CDK5R2)] (Ruiz de Porras et al., 2019). In the present study, we demonstrated that Cdk5 expression was significantly upregulated in CRC (Supplementary Figure S1), while knockdown of Cdk5 led to a significant reduction in the migration and invasive abilities of the DLD-1 and SW480 cells (Supplementary Figures S2–S4). Animal studies also demonstrated that knockdown of Cdk5 inhibited tumor growth (Supplementary Figure S5), while levels of cyclin D1 and c-Myc expression were markedly decreased in the Cdk5 knockdown group vs. control group. Cyclin D1 and c-Myc are target genes of the Wnt/β-catenin signaling pathway, as previously reported in human HT29 CRC cells harboring mutant Adenomatous polyposis coli gene (APC) alleles (He et al., 1998). The majority of sporadic CRCs have mutations in genes involved in the Wnt/β-catenin signaling pathway (Marra et al., 2013), and these mutations frequently occur in the earliest neoplasms. These findings suggest that the Wnt/β-catenin signaling pathway may act as an important gatekeeper to prevent CRC (Giles et al., 2003). When aberrantly activated, the Wnt/β-catenin signaling pathway can result in the accumulation of β-catenin in the cytoplasm and enhancement of its translocation to the nucleus, where it can trigger the β-catenin/T-cell factor/lymphoid enhancer factor transcriptional machinery, resulting in upregulation of target genes (e.g., c-Myc, cyclin D1, matrix metalloproteinase-7) (Humphries and Wright, 2008). In this study, we assessed the levels of these proteins by WB and found that Cdk5 knockdown inhibited the expression of β-catenin, cyclin D1, and c-Myc in DLD-1 cells. Based on these findings, we propose that Cdk5 is involved in the regulation of CRC cell proliferation and migration *via* the Wnt/β-catenin signaling.

In this study, we explored the mechanisms whereby *F. nucleatum* promotes the proliferation and migration of CRC cells. Initially, we examined the correlation between the abundance of *F. nucleatum* and Cdk5 in CRC. The results showed that Cdk5 expression was significantly higher in human CRC tissues than in histologically normal control specimens, and the abundance *F. nucleatum* was positively correlated with Cdk5 expression. Furthermore, we showed that Cdk5 expression was significantly elevated in response to *F. nucleatum*, whereas knockdown of Cdk5 abrogated *F. nucleatum-*mediated proliferation and migration of CRC cells, suggesting the direct role of Cdk5 in the effect of *F. nucleatum*.

A number of recent studies have indicated that *F. nucleatum* is associated with the development of CRC (Yang et al., 2017; Yu et al., 2017). Consistent with these previous studies, the high-throughput sequencing in our study revealed that the abundance of *Fusobacterium* increased in human CRC stool samples. It has been reported that *F. nucleatum* can activate the Wnt/β-catenin signaling pathway, thereby promoting the growth of CRC cells (Mima et al., 2016). In cancer cells, Annexin A1 expression levels are elevated, and *F. nucleatum* activates β-catenin through the FadA–E-cadherin–Annexin A1–β-catenin complex, thereby accelerating tumor progression (Rubinstein et al., 2019). In addition, it has been found that (Chen et al., 2017; Wu et al., 2018) invasive *F. nucleatum* can activate β-catenin signal in CRC through the toll-like receptor 4 (TLR4)/phospho-p21-activated kinase 1 (P-PAK1)/P-β-catenin S675 cascade, leading to a significant increase in the nuclear translocation of β-catenin and thus promoting the occurrence of intestinal tumors. Based on these findings, we investigated the potential role of Cdk5 in promoting the *F. nucleatum-*mediated proliferation and migration of CRC cells, including DLD-1 and SW480 cells. Interestingly, *F. nucleatum* upregulated Cdk5 expression and activated the Wnt/β-catenin signaling pathway *in vitro*, while knockdown of Cdk5 abrogated these effects, indicating that *F. nucleatum* can activate the Wnt/β-catenin signaling pathway through upregulation of Cdk 5 expression. In addition, our ongoing study suggested that *F. nucleatum*-derived metabolites stimulate the Wnt/β-catenin signaling pathway through upregulating Cdk5 (data not shown). However, the exact mechanism of action how *F. nucleatum* stimulates CRC through the Cdk5 and Wnt/β-catenin signaling pathway will need further study in the future.

STAT3 is a well-known oncogene, and its phosphorylation is necessary for its role. Following phosphorylation, p-STAT3 promotes its translocation from the cytoplasm to the nucleus, where it plays a regulatory role in modulating the transcription of target genes. Importantly, these genes modulate multiple cellular processes related to the development and progression of cancer, including proliferation, migration, and differentiation (Huang et al., 2011). The results of the present study demonstrated that *F. nucleatum* led to an increase in the levels of p-STAT3 in DLD-1 cells in support of *F. nucleatum*-mediated promotion of CRC cell proliferation. It has been noted that persistent infections and chronic inflammation are contributing factors of various cancers (e.g., colitis-associated cancer, skin cancer, hepatocellular carcinoma). In fact, extensive immune cell infiltration with high levels of mediators of inflammation has been reported in the tumor microenvironment (Grivennikov et al., 2010; Grivennikov and Karin, 2011; Elinav et al., 2013). *F. nucleatum* infection enhanced the tumor growth of CRC in a TLR4-dependent manner, and the effect involved activation of the IL-6/p-STAT3/c-MYC signaling pathway (Chen et al., 2018). In our study, the mRNA levels of a number of inflammatory factors such as IL-6, IL-8, COX-2, and TNF-α were significantly higher in human CRC tissues than in matched normal controls and in cells treated with *F. nucleatum* vs. control cells. These findings indicated that STAT3 stimulated intracellular inflammation which mediated by IL-6, may caused by *F. nucleatum* infection *via* TLR4 dependence, facilitating the development and progression of CRC.

It has been noted that both Wnt/β-catenin and STAT3 signaling pathways are closely related to tumors and have synergism with the occurrence and progression of tumor, in which STAT3 upregulates the protein expression and transcriptional activity of β-catenin in breast cancer (Armanious et al., 2010). Kawada et al. (2006) have demonstrated that activation of STAT3 in CRC causes the accumulation of β-catenin in the nucleus, thus accelerating tumor progression. Based on these existing results, we propose a hypothetical model for *F. nucleatum*-mediated promotion of cell proliferation and migration of CRC cells (Figure 6E). In this proposed model, *F. nucleatum*-derived metabolites stimulate the Wnt/β-catenin signaling pathway through upregulating Cdk5. In addition, *F. nucleatum* activates IL-6/STAT3 in a TLR4-dependent manner to stimulate Wnt/β-catenin signaling pathway. However, the exact mechanism of action how *F. nucleatum* stimulates CRC through the Cdk5 and Wnt/β-catenin signaling pathway will need further study in the future.

Taken together, the results of the experiments performed in human tissues and cells showed that *F. nucleatum* promoted the proliferation and invasion of CRC by orchestrating a molecular network in which Cdk5 has a direct role in activating Wnt/β-catenin signaling. These new findings may provide a better understanding of the pathogenesis of CRC. Future in-depth investigations of *F. nucleatum-*associated molecular pathways may also further the development of treatment for CRC.

## Data Availability Statement

The original contributions presented in the study are included in the article/Supplementary Materials, further inquiries can be directed to the corresponding author/s.

## Ethics Statement

The animal study was reviewed and approved by the Institutional Animal Committee of Wenzhou Medical University.

## Author Contributions

XL, YL, and FJ contributed to the study concept and design. LJ and FJ contributed to the specimen collection. JH, TY, XF, and LL contributed to the analysis and interpretation of data and statistical analysis. JH, TY, XF, and SX contributed to the animal experiments. XL and YL contributed to the drafting of the manuscript. All authors contributed to the article and approved the submitted version.

## Conflict of Interest

The authors declare that the research was conducted in the absence of any commercial or financial relationships that could be construed as a potential conflict of interest.
