# Supplementary material for: Fusobacterium nucleatum Promotes the Progression of Colorectal Cancer Through Cdk5-Activated Wnt/β-Catenin Signaling
Source: Front Microbiol. 2021 Jan 6;11:545251. doi: 10.3389/fmicb.2020.545251 (PMC7815597; doi:10.3389/fmicb.2020.545251)
Supplement: Supplementary file 1 [file Table_1.DOCX]

***F.nucleatum* facilitates the proliferation and migration of colorectal cancer cells through Wnt/β-catenin signaling via the upregulation of cyclin dependent protein kinase 5**

**Table S1.** List of primers used.

| Target | | Sequence 5’-3’ | Purpose |
| --- | --- | --- | --- |
| *F. nucleatum* | Forward | 5’- TTCAATAAAAGTGGCAGGTCAAG-3’ | qPCR |
|  | Reverse | 5’- TAACAACACATGCAGGTCAATGG-3’ |  |
| Cdk5 | Forward | 5’- ATGCAGAAATACGAGAAACTGG -3’ | qPCR |
|  | Reverse | 5’- GACGATGTTCTTGTGCTTCA -3’ |  |
| GAPDH | Forward | 5’- TGGACTCCACGACGTACTCAG -3’ | qPCR |
|  | Reverse | 5’- ACATGTTCCAATATGATTCCA -3’ |  |
|  | Reverse | 5’-GACAGAAGGGCATCTAGCGAC-3’ |  |
| COX-2 | Forward | 5’- TGAGCATCTACGGTTTGCTG -3’ | qPCR |
|  | Reverse | 5’- TGCTTGTCTGGAACAACTGC -3’ |  |
| IL6 | Forward | 5’- CAATGAGGAGACTTGCCTGGTG -3’ | qPCR |
|  | Reverse | 5’- GGTTGGGTCAGGGGTGGTTA -3’ |  |
| IL10 | Forward | 5’- CATCAAGGCGCATGTGAACT -3’ | qPCR |
|  | Reverse | 5’- GATGTCAAACTCACTCATGGCTTT -3’ |  |
| TNF-α | Forward | 5’- ATGTTGTAGCAAACCCTCAAGC -3’ | qPCR |
|  | Reverse | 5’- TGTGGGTGAGGAGCACAT -3’ |  |
|  | Reverse | 5’-GGGGATGATTTGCAGGTATGT-3’ |  |
|  | Reverse | 5’-GAGGAGGTTGACTTTCTCCTGG-3’ |  |
| 16S DNA | Forward | 5’-CGTCAGCTCGTGYCGTGAG-3’ | qPCR |
|  | Reverse | 5’- CGTCRTCCCCRCCTTCC -3’ |  |


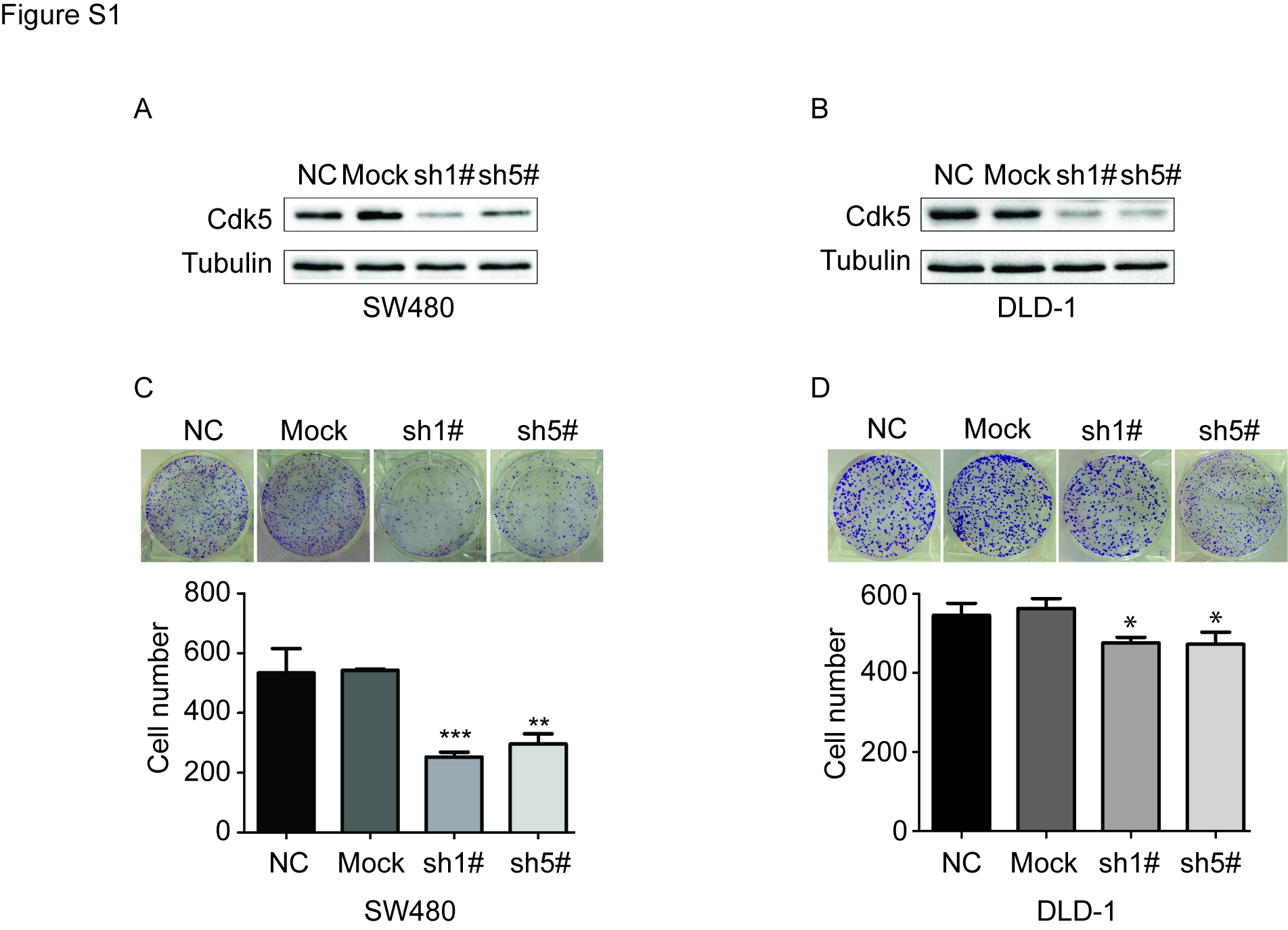


Figure S1. Cdk5 knockdown inhibits the proliferation of DLD-1 and SW480 cells.

(A) The levels of Cdk5 were measured by Western blotting in Cdk5-depleted SW480 cells. (B) The levels of Cdk5 were measured by Western blotting in Cdk5-depleted DLD-1 cells. (C) Depletion of endogenous Cdk5 inhibited the proliferation of SW480 cells. ***P* < 0.01 and ****P* < 0.001 are compared to the control. (D) Depletion of endogenous Cdk5 inhibited the proliferation of DLD-1 cells. **P* < 0.05 is compared to the control.


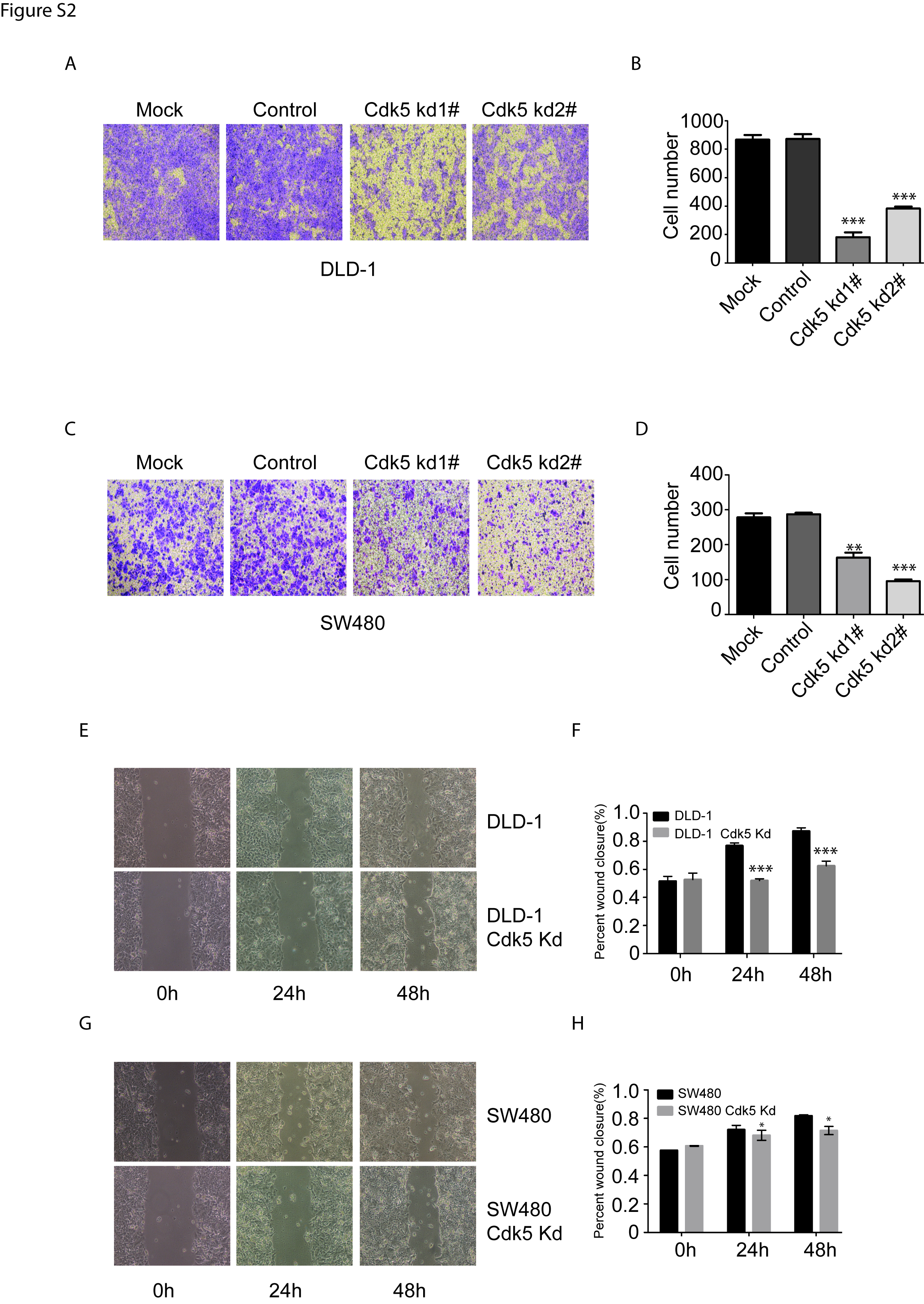


Figure S2: Cdk5 knockdown inhibited migration and invasion of DLD-1 and SW480 cells. (A) Depletion of endogenous Cdk5 inhibited the invasive ability of DLD-1 cells. (B) Quantification of invasion assays. Data are expressed as mean ± S.E.M. from at least three independent experiments. ***P<0.001 vs. control. (C) Depletion of endogenous Cdk5 inhibited the invasive ability of SW480 cells. (D) Quantification of invasion assays. Data are expressed as mean ± S.E.M. from at least three independent experiments. **P<0.01 and***P<0.001 vs. control. (E) Wound-healing assays in DLD-1 cells (×400). (F) Quantification of wound-healing assays. Data are expressed as mean ± S.E.M. from at least three independent experiments. ***P<0.001 vs. control. (G) Wound-healing assays in SW480 cells (×400). (H) Quantification of wound-healing assays. Data are expressed as mean ± S.E.M. from at least three independent experiments. *P<0.05 vs. control.


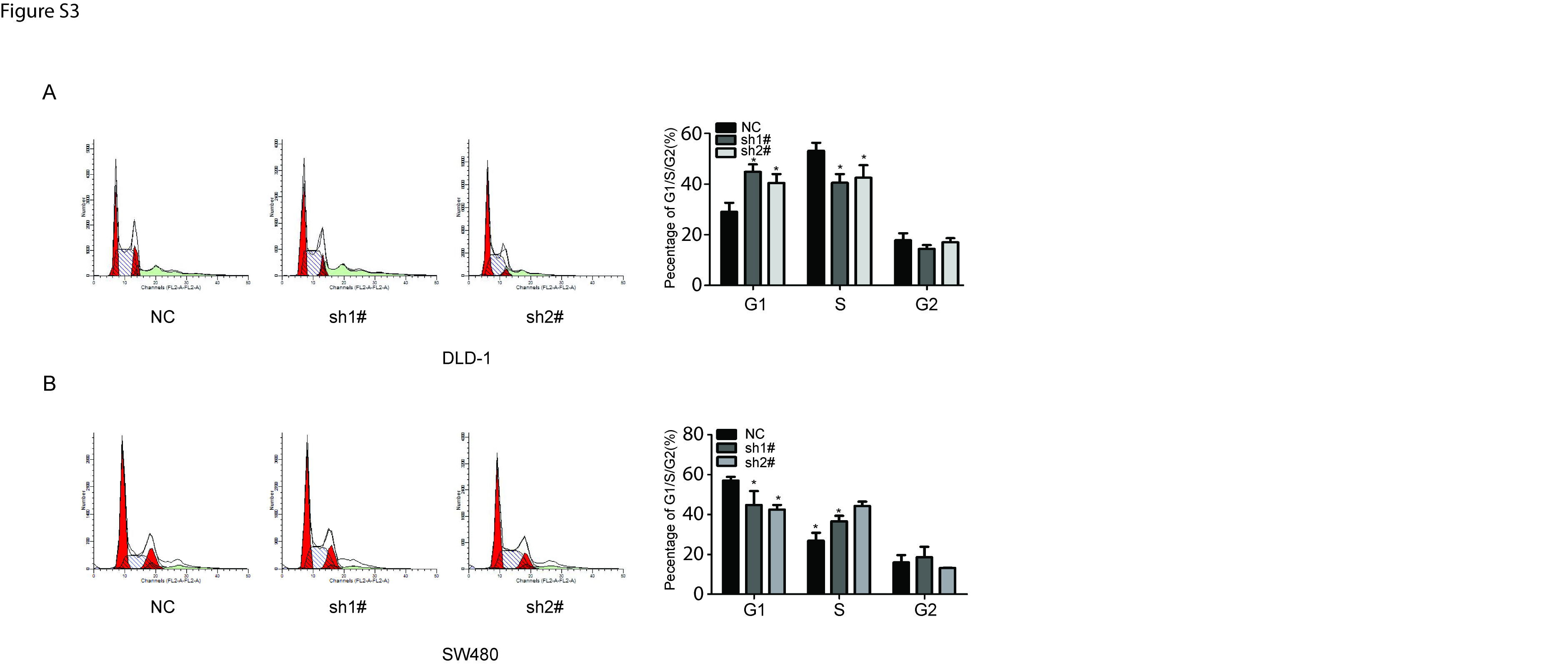


Figure 3: Cdk5 knocknock downown blocks SW480 and DLD-1 cell cycle. (A) flow cytometry to detect DLD-1 cell cycle distribution. (B) flow cytometry to detect SW480 cell cycle distribution. Each experiment was performed in triplicate. *P < 0.05.


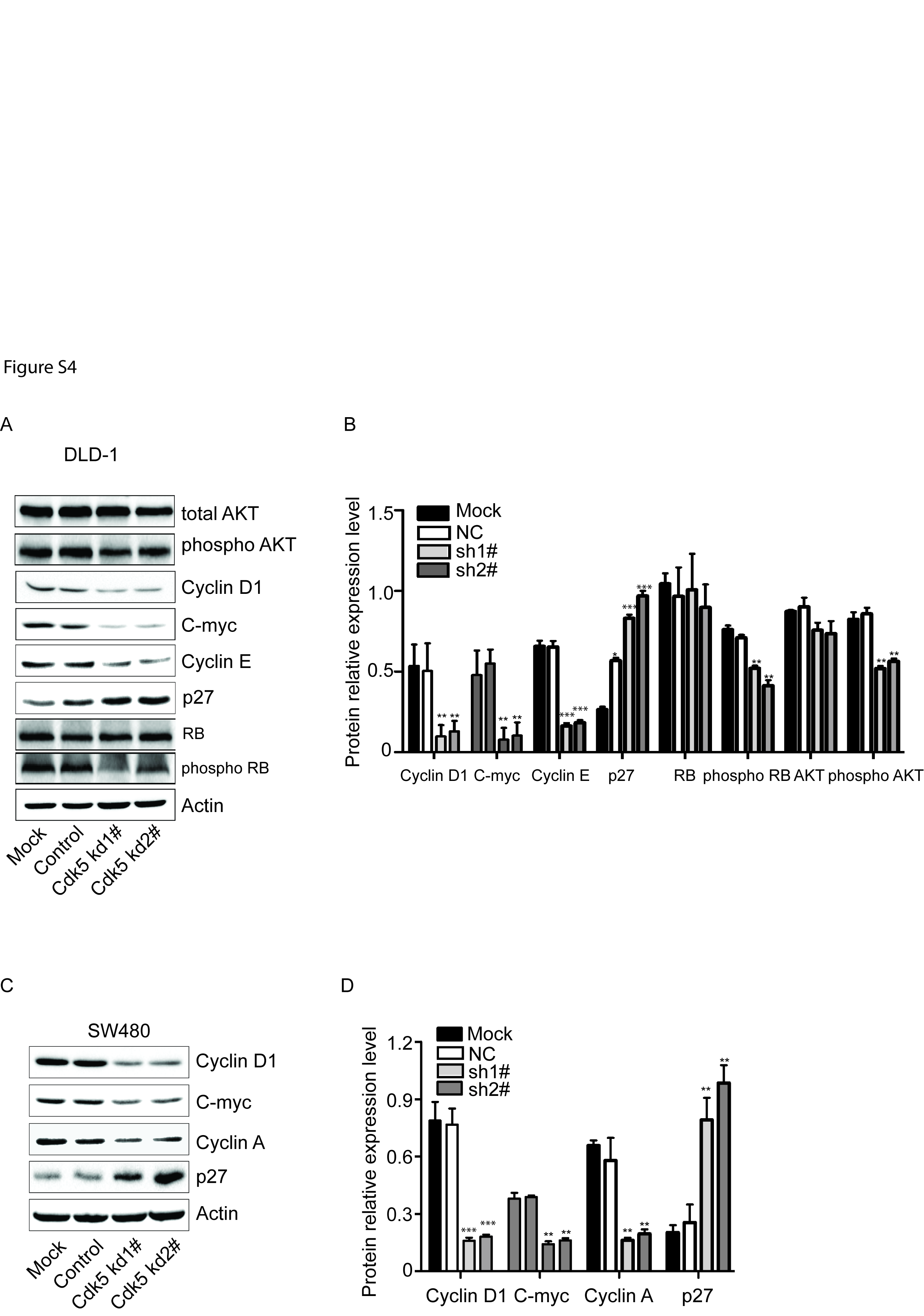


Figure. S4 Cdk5 knockdown effects on expression levels of cell cycle signaling pathway associated proteins in DLD-1 and SW480 cell lines. (A) The expression levels of AKT, phosphor AKT, cyclinD1, C-myc, cyclinE, p27, Rb and phospho Rb were measured by Western blotting in DLD-1 cells. (B) The relative protein expression levels of AKT, phosphor AKT, cyclinD1, C-myc, cyclinE, p27, Rb and phospho Rb in DLD-1 cells. **P < 0.01 and ***P < 0.001 are compared to the control. (C) The expression cyclinD1, C-myc, cyclinA and p27 were measured by Western blotting in SW480 cells. (D) The relative protein expression levels of cyclinD1, C-myc, cyclinA and p27 in SW480 cells. **P < 0.01 and ***P < 0.001 are compared to the control.


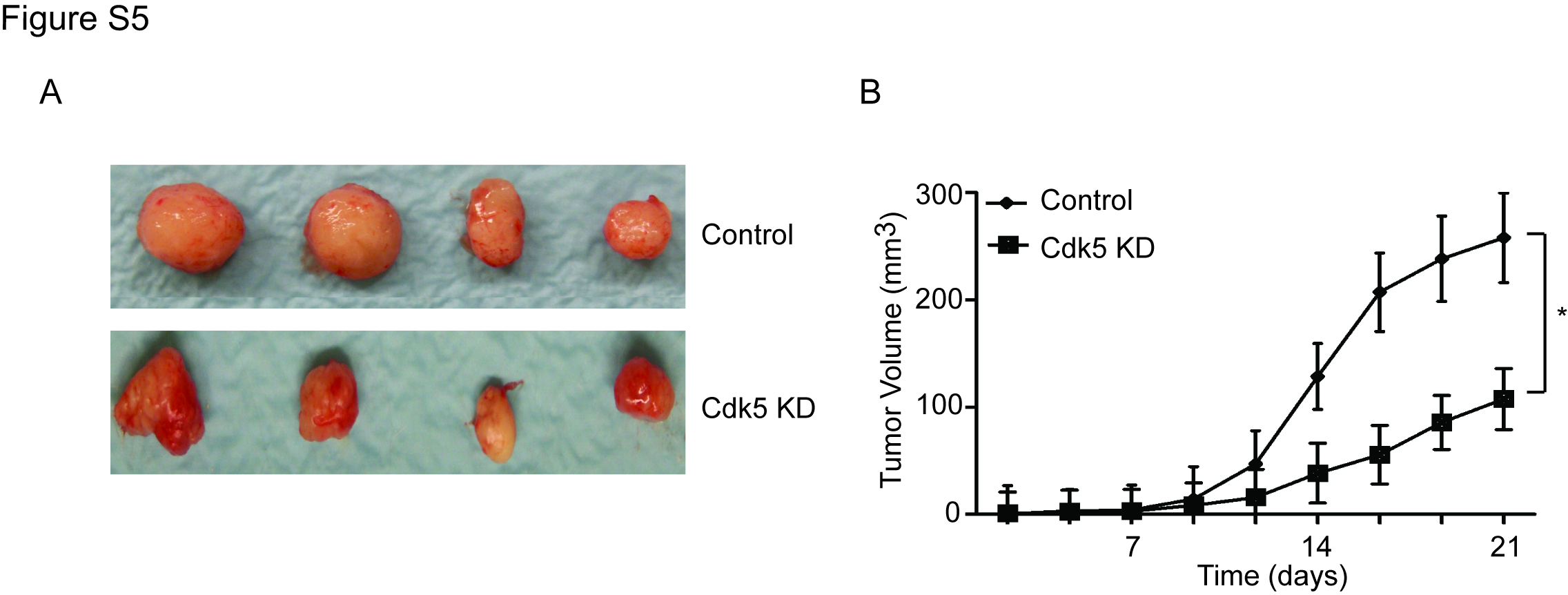


**Figure** S5 Cdk5 knockdown suppressed tumor growth in a xenograft model.

BALB/c nude mice (5 to 6 weeks old) were injected subcutaneously with 1×10^6^ shRNA control, Cdk5-depleted DLD-1cells. Tumor size was measured daily or every other day with calipers, and tumor volumes were calculated using the formula: Volume = (width)^2^ × length/2. Cdk5 knockdown inhibited tumor growth (A), and tumor volumes (B). **P* < 0.05 is compared to the control.
